# Editorial: Composite and Functionalized Hydrogels: Implications for Improved and Biological Properties in Tissue Engineering

**DOI:** 10.3389/fbioe.2020.636575

**Published:** 2021-01-12

**Authors:** Piergiorgio Gentile, Elvira De Giglio, Jons Gunnar Hilborn, Kee Woei Ng

**Affiliations:** ^1^School of Engineering, Newcastle University, Newcastle upon Tyne, United Kingdom; ^2^Department of Chemistry, University of Bari Aldo Moro, Bari, Italy; ^3^Department of Chemistry, Uppsala University, Uppsala, Sweden; ^4^School of Materials Science and Engineering, Nanyang Technological University, Singapore, Singapore; ^5^Environmental Chemistry & Materials Centre, Nanyang Environment and Water Research Institute (NEWRI), Singapore, Singapore; ^6^Center for Nanotechnology and Nanotoxicology, Harvard T.H. Chan School of Public Health, Harvard University, Boston, MA, United States

**Keywords:** hydrogels, functionalisation, composite, mechanical propedies, biochemical tuning

This Research Topic collects different contributions on the design and preparation of application-driven 3D composite and/or functionalized cell-laden hydrogels for the Tissue Engineering field. Owing to their high-water content as well as their amenability to mechanical and biochemical tuning, many hydrogels lend themselves as extracellular matrix mimetics. However, to benefit from their potential, some limitations remain to be overcome, as these still do not match the mechanical or biological properties of native tissues. In this Research Topic, we present new insights on current strategies explored to enhance the application of the hydrogel to improve the mechanical properties as well as functionalization to mimic the natural extracellular environment better.

The first article of this Research Topic (Zhao et al.) introduces the recent advances in using composite hydrogels laden with cells as biomimetic tissue- or organ-like constructs and as matrices for multi-cell type organoid cultures. The authors highlight that specifically designed hydrogels are relevant tissue-like matrices to support the development of 3D *in vitro* models possessing features of the native extracellular matrix (ECM). Furthermore, composite hydrogels have gained increasing attention for further enhancement of the matrix relevance or to impart specific functionalities. Examples are the incorporation of additional ECM components to enhance e.g., mechanical properties, to incorporate drugs to control cell fates, biomolecules to affect biological activities or any combinations of the above. Moreover, the authors present a critical overview on the latest composite hydrogels that incorporate biological cues, nanomaterials, and combinations of biopolymers including Soft Network Composites (SNCs) and Interpenetrating Networks (IPNs). Thus, composite hydrogels provide a platform for designer matrices to be produced in the laboratory that are rapidly approaching the final properties desired by optimizing the selection of the constitutes, composition ratio, polymers, engrafted functional groups, and the interaction of different materials (physical or chemical bonding). However, challenges on the validation of reliability and reproducibility of 3D tissue models remain for their future adoption as standard testing platforms and clinical translation.

Recently, the use of composite hydrogels for mechano-transduction studies has attracted attention from the scientific community, due to their combined bio functionality and mechanical properties. Notably, the interest in understanding how mechanical signals influence cell behavior is shared across different disciplines such as cancer and developmental biology, regenerative medicine, tissue engineering, and clinical fields such as orthopedics. As an example, mechanical loading is a known contributor to intervertebral disc (IVD) degeneration. In this regard, Cambria et al. investigated how to improve the biological properties of agarose, which is a standard biomaterial suitable for cartilage and intervertebral disc regeneration. However, it lacks in adhesion motifs and necessary cell-matrix interaction for mechano-transduction. The authors propose a novel agarose-collagen composite hydrogel that combines the mechanical properties of agarose and the biofunctionality of collagen type I simultaneously. The addition of collagen at different concentration can significantly influence the storage and loss modulus of the obtained composite hydrogels. Specifically, the blended gels containing a lower collagen concentration (2 mg/mL) display similar mechanical properties to agarose. Furthermore, embedded nucleus pulposus (NP) cells are more than 80% viable with reduced proliferation and a round morphology typical of NP cells *in vivo*. The authors also evidence a significantly increased gene expression of collagen types I and II and aggrecan in composite hydrogels from day 1 to 7, further resulting in a significantly superior GAG/DNA ratio compared to agarose gels at day 7. Finally, for their mechano-transduction ability, the composite hydrogels show around five-fold higher focal adhesion kinase phosphorylation (pFAK/β-tubulin) when not compressed, and increased pFAK/FAK values 10 min after compression. This represents one of the first steps in the integrin-mediated mechano-transduction mechanisms, allowing these agarose-collagen hydrogels to be explored for complex loading scenarios in a highly reproducible system.

Another exciting strategy reported in this Research Topic consists of the manufacturing of functionalized hydrogels, to tune the mechanical properties of the cellular microenvironment that is key in modulating cell function. Indeed, many pathophysiological processes are associated with variations in ECM stiffness. In their work Tirella et al. investigate the role of the Lysyl oxidase (LOx), an enzyme involved in several ECM-stiffening processes, on the final mechanical properties of preparing poly(ethylene glycol) (PEG)-based hydrogels. Notably, the elastic moduli are in the range of 0.5–4 kPa after a first photopolymerization step, while they show a stiffness increase up to 0.5 kPa after incubation with the LOx enzyme. The obtained gel formulation embedded with HepG2 cells is proposed as bioink to form 3D *in vitro* models to mimic hepatic tissue. In this work, hepatic functional markers are measured up to 7 days of culture, suggesting further use of such 3D models to study cell mechanobiology and response to dynamic variation of hydrogels stiffness.

Finally, the optimization of the formulation of composite and functionalized hydrogels is a crucial component for the successfully manufacturing of bioprinted constructs. In this sense, the synthesis of hydrogels, their crosslink, and physicochemical properties (e.g., viscosity, mechanical properties), as well as the bioprinting process, represent different challenges for the scientific community. Although multiple combinations of materials and processing parameters have been used for several applications, the relationship between good printability of bioinks and suitability in medical/clinical use of bioprinted constructs is still unclear. For this reason, Sanchez et al. propose the application of the PRISMA methodology to extract from the literature information on materials, hydrogel synthesis, bioprinting process, and tests performed on bioprinted constructs. In their review, the authors retrieve 1,774 papers from different scientific databases (e.g., PUBMED, WOS, and SCOPUS), and they select 118 articles for their analysis. This systematic review aims to investigate the materials used and their influence on the bioprinting parameters and to compare the mechanical and cellular behavior of those bioprinted structures. Final considerations are also reported on the importance of the correct definition of the following parameters involved in bioprinting: (i) concentration and viscosity of materials; (2) bed temperature and printing speed in addition to the cartridge temperature and printing pressure; (3) printability window; (4) information on the crosslinking reaction. These parameters are crucial for validating the reliability and standardization of the bioprinted hydrogels.

It is clear from the articles received for this Research Topic that composite hydrogels are a highly versatile platform that allows multiple strategies to re-create a myriad of microenvironments for realistic 3D culture models that mimic native tissues. While the relevance of adapting these to produce transplantable tissues and organs is debatable, it is anticipated that composite hydrogels will become the modus operandi for generating validated, reproducible 3D models for screening of bioactives and for more accurate biological explorations *in vitro*.

## Author Contributions

All authors listed have made a substantial, direct and intellectual contribution to the work, and approved it for publication.

## Conflict of Interest

The authors declare that the research was conducted in the absence of any commercial or financial relationships that could be construed as a potential conflict of interest.

